# 821. Positive Follow-Up Blood Culture in Candidemia and Ocular Involvement

**DOI:** 10.1093/ofid/ofad500.866

**Published:** 2023-11-27

**Authors:** Hamail Iqbal, Dejan Nikolic, Carlo Foppiano Palacios

**Affiliations:** Cooper Medical School of Rowan University, Summit, New Jersey; Cooper University Health Care, Camden, New Jersey; Cooper University Health Care, Camden, New Jersey

## Abstract

**Background:**

Candidemia can lead to ocular complications, including endophthalmitis and chorioretinitis. Prompt management of such ocular diseases is paramount due to the risk of blindness. Management of candidemia usually includes a dilated ophthalmic exam; however, there are concerns regarding the utility of performing ophthalmic exams in all patients due to a low rate of ocular complications and lack of changes in medical management. Thus, there is a need for prognostic factors suggestive of ocular involvement that warrant a thorough ophthalmic examination. We sought to evaluate if positive repeat blood cultures are associated with ocular involvement in candidemia.

**Methods:**

A single-center, retrospective chart review was conducted at an academic medical center. All candidemia cases from 2017 to 2022 were analyzed for demographics, culture results, treatment, inpatient ophthalmic exam findings, and mortality at discharge, at 30 days, and at 90 days. Descriptive statistics and Chi-Square tests were performed on the collected data.

**Results:**

104 episodes of candidemia among 84 patients were included: the mean age was 54 years, 55% were male, 74% were Black, and 13.1% were Latino. The most common Candida species isolated was C. albicans (37%) and most patients were treated with micafungin (51%). 12% had visual symptoms and 84% underwent ophthalmic exams. 15% of the exams were concerning for ocular involvement: 5% endophthalmitis, 5% chorioretinitis, and 5% other infectious findings (Roth spots and other retinal lesions). There was no significant association between repeat positive blood culture results and development of endophthalmitis (p = 1.00), chorioretinitis (p = 0.93), or any ocular involvement (p = 0.97). Furthermore, no significant associations were found between repeat blood culture and death at discharge, 30 days, or 90 days.

**Conclusion:**

No significant associations were found between repeat positive blood culture and ocular involvement in the setting of candidemia. Other indicators of ocular involvement in candidemia are needed to assess patients who would benefit from ophthalmic exam.

**Disclosures:**

**All Authors**: No reported disclosures

